# A Novel TREM2 Small Molecule Agonist MTX46943 Re‐Programs Human Microglia and Reduces Amyloid Pathology *In Vivo*

**DOI:** 10.1002/alz70859_100777

**Published:** 2025-12-25

**Authors:** Manuela Polydoro, Ivana Geric, Jin Zheng, Tina Sommer Bisgaard, Ashley Comer, Laura Sans, Ashley Lu, Joachim Vilstrup, Leen Wolfs, Arya Nair, Morten S Andersen, Ninna Frederiksen, Peter Flagstad, Maria Dalby, Rita Balice‐Gordon, Niels Plath

**Affiliations:** ^1^ Muna Therapeutics, Copenhagen, Capital Region Denmark; ^2^ Muna Therapeutics, Leuven Belgium; ^3^ Muna Therapeutics, Copenhagen Denmark

## Abstract

**Background:**

Microglia function as brain‐resident innate immune cells that maintain brain health and play a critical role in resolving Alzheimer´s Disease (AD) pathology. TREM2 modulates microglia responses to neutralize misfolded proteins and ultimately restore neuronal function. MTX46943 is a novel, potent, selective, safe and brain‐penetrant TREM2 small molecule agonist developed for the treatment of early AD. We show here key features of the mechanism and efficacy of MTX46943 in activating microglia and reducing AD pathology following chronic treatment *in vivo*.

**Method:**

TREM2 agonist‐induced receptor complex dynamics and downstream functional effects were assessed *in vitro* in CHO, HEK, THP‐1 and hIPSC derived microglia cells by nanoBiT, Western blot, alphaLISA, cell migration and phagocytosis assays. Differences in receptor activation between agonist modalities were studied by comparing MTX46943 with TREM2 agonist antibodies. The impact of MTX46943 on amyloid pathology *in vivo* was tested with 3‐month treatment of 5xFAD // hTREM2 knock‐in mice followed by human microglia and brain tissue isolation for qPCR, single‐cell RNA sequencing and immunostaining analyses.

**Result:**

MTX46943‐mediated TREM2 activation leads to TREM2/DAP12 receptor complex formation and stabilizes surface location required for downstream signaling and microglia activation. These effects are differentiated from TREM2 agonistic antibodies and are required for downstream microglial activation. *In vivo*, human TREM2 is expressed by pathology‐associated microglia and induced by amyloid plaques. Chronic, systemic treatment with MTX46943 leads to re‐programming of microglia in the presence of amyloid pathology which in turn leads to reduction of neurotoxic amyloid beta species. Effects were observed at compound exposure levels in the brain consistent with MTX46943 *in vitro* potency.

**Conclusion:**

Muna Therapeutics is advancing MTX46943, a selective, potent, safe and brain exposed small molecule TREM2 agonist into clinical studies for the treatment of early AD. We demonstrate here key differentiated aspects of the mechanism by which MTX46943 targets TREM2 and induces microglial re‐programming via receptor complex formation and stabilization. Chronic treatment of MTX46943 robustly and significantly reduces brain amyloid pathology at doses shown to promote peripheral and central biomarker responses. The present studies collectively support the best‐in‐class therapeutic potential of MTX46943 for the treatment of Alzheimer´s disease.

